# *Pyrococcus furiosus* Argonaute-mediated porcine epidemic diarrhea virus detection

**DOI:** 10.1007/s00253-023-12919-0

**Published:** 2024-01-15

**Authors:** Yu Zhao, Changyu Zhou, Boyan Guo, Xin Yang, Hongning Wang

**Affiliations:** 1 Animal Disease Prevention and Food Safety Key Laboratory of Sichuan Province, College of Life Sciences, Sichuan University, Chengdu, China; 2 Key Laboratory of Bio-Resource and Eco-Environment of Ministry of Education, College of Life Sciences, Sichuan University, Chengdu, China

**Keywords:** Porcine epidemic diarrhea virus, Diagnosis technology, RAA, PfAgo

## Abstract

**Abstract:**

Porcine epidemic diarrhea virus (PEDV), an enteric coronavirus, induces severe vomiting and acute watery diarrhea in unweaned piglets. The pig industry has suffered tremendous financial losses due to the high mortality rate of piglets caused by PEDV. Consequently, a simple and rapid on-site diagnostic technology is crucial for preventing and controlling PEDV. This study established a detection method for PEDV using recombinase-aided amplification (RAA) and *Pyrococcus furiosus* Argonaute (PfAgo), which can detect 100 copies of PEDV without cross-reactivity with other pathogens. The entire reaction of RAA and PfAgo to detect PEDV does not require sophisticated instruments, and the reaction results can be observed with the naked eye. Overall, this integrated RAA-PfAgo cleavage assay is a practical tool for accurately and quickly detecting PEDV.

**Key points:**

*• PfAgo has the potential to serve as a viable molecular diagnostic tool for the detection and diagnosis of viral genomes*

*• The RAA-PfAgo detection technique has a remarkable level of sensitivity and specificity*

*• The RAA-PfAgo detection system can identify PEDV without needing advanced equipment*

## Introduction

Porcine diarrhea in the global swine industry is most commonly caused by PEDV, and PEDV is an enveloped virus that belongs to the family Coronaviridae and the genus Alphacoronavirus (Li et al. 2021a). PEDV infects pigs of all ages, causing vomiting, acute watery diarrhea, dehydration, and weight loss. The mortality rate of piglets reaches 100% (Tian et al. 2021). Since the discovery of PED in the UK in 1971 (Chasey and Cartwright 1978), it has spread to many countries, including the USA (Huang et al. 2013), Europe (Jung and Saif 2015), and China (Sun et al. 2012), where it has resulted in significant economic losses for the pig industry. However, PEDV is frequently co-infected with other coronaviruses in pig farms, including porcine delta coronavirus (PDCoV) and transmissible gastroenteritis virus (TGEV) (Ding et al. 2020a), which can cause similar clinical symptoms and make differential diagnosis challenging. Consequently, developing novel, simple, and effective approaches for detecting PEDV is urgently needed.

Currently, numerous techniques for detecting PEDV have been reported, including conventional virus isolation (Valkó et al. 2019), enzyme-linked immune sorbent (ELISA) (Gerber et al. 2014), and molecular biological techniques (Kim et al. 2007). Virus isolation and identification is a laborious process that takes a lot of time and necessitates specialized equipment and personnel; ELISA has been widely used to detect the level of antibodies in serum, but it was not a good choice for early diagnosis of viral infection as antibody generation required a few days, it was also laborious and time-consuming (Pewlaoo et al. 2022; Wang et al. 2020). Several molecular biological techniques commonly employed for PEDV diagnostics are quantitative polymerase chain reaction (qPCR) (Pan et al. 2020), recombinase polymerase amplification (RPA) (Li et al. 2021b), and loop-mediated isothermal amplification (LAMP) (Yu et al. 2015). Nevertheless, these technologies possess many drawbacks, including requiring skilled workers or costly and intricate equipment for qPCR (Yu et al. 2015), alongside elevated rates of false positives when solely employing RPA (Qian et al. 2023; Qin et al. 2022; Xu et al. 2022), complex primer design for LAMP (Kim et al. 2021; Li et al. 2022). The introduction and development of CRISPR diagnostic technology have provided us with an improved option for addressing the issues mentioned above. Bacteria and archaea possess adaptive immune systems known as clustered, regularly interspaced short palindromic repeat (CRISPR)-Cas systems. Currently, the CRISPR system consists primarily of Cas9, Cas12, Cas13, and Cas14. Cas9 and Cas12a are used primarily to cut double-stranded DNA, and Cas14a and Cas13a are primarily used to cut single-stranded DNA and single-stranded RNA, respectively. Currently, these nucleases are utilized in various virus detection techniques (Ding et al. 2020b; Kellner et al. 2019; Mali et al. 2013; Yang et al. 2021a). Current PEDV detection methods based on Cas12a are fast, specific, sensitive, and reliable; these attributes will be essential in the future development of point-of-care testing (POCT) molecular diagnostic technologies for viral infectious diseases (Liu et al. 2022; Qian et al. 2022; Yang et al. 2021b). However, the CRISPR system also has some limitations. Firstly, Cas proteins can function only at room temperature and become inactive at high temperatures, thereby influencing cleavage activity. Cas proteins require a section of gRNA guidance to cleave the target nucleic acid; however, RNA is susceptible to degradation, which makes storage and transport difficult. Additionally, gRNA synthesis is quite expensive.

A typical DNA-guided endonuclease in pAgos is the archaea *Pyrococcus furiosus* Argonaute (PfAgo), which cleaves the phosphodiester bond between the 10th and 11th bases of the target DNA from the 5′ end (Swarts et al. 2015). Compared to the CRISPR system, PfAgo only requires gDNA guidance to cut single-stranded DNA. PfAgo can withstand high temperatures, making it more suitable for transportation and preservation. PfAgo also provides greater gDNA selection versatility because it does not necessitate a PAM (protospacer adjacent motif) site. Applications of the PfAgo to molecular detections have emerged in recent years (He et al. 2019; Wang et al. 2021; Yang et al. 2023; Zhao et al. 2022).

**Fig. 1:**
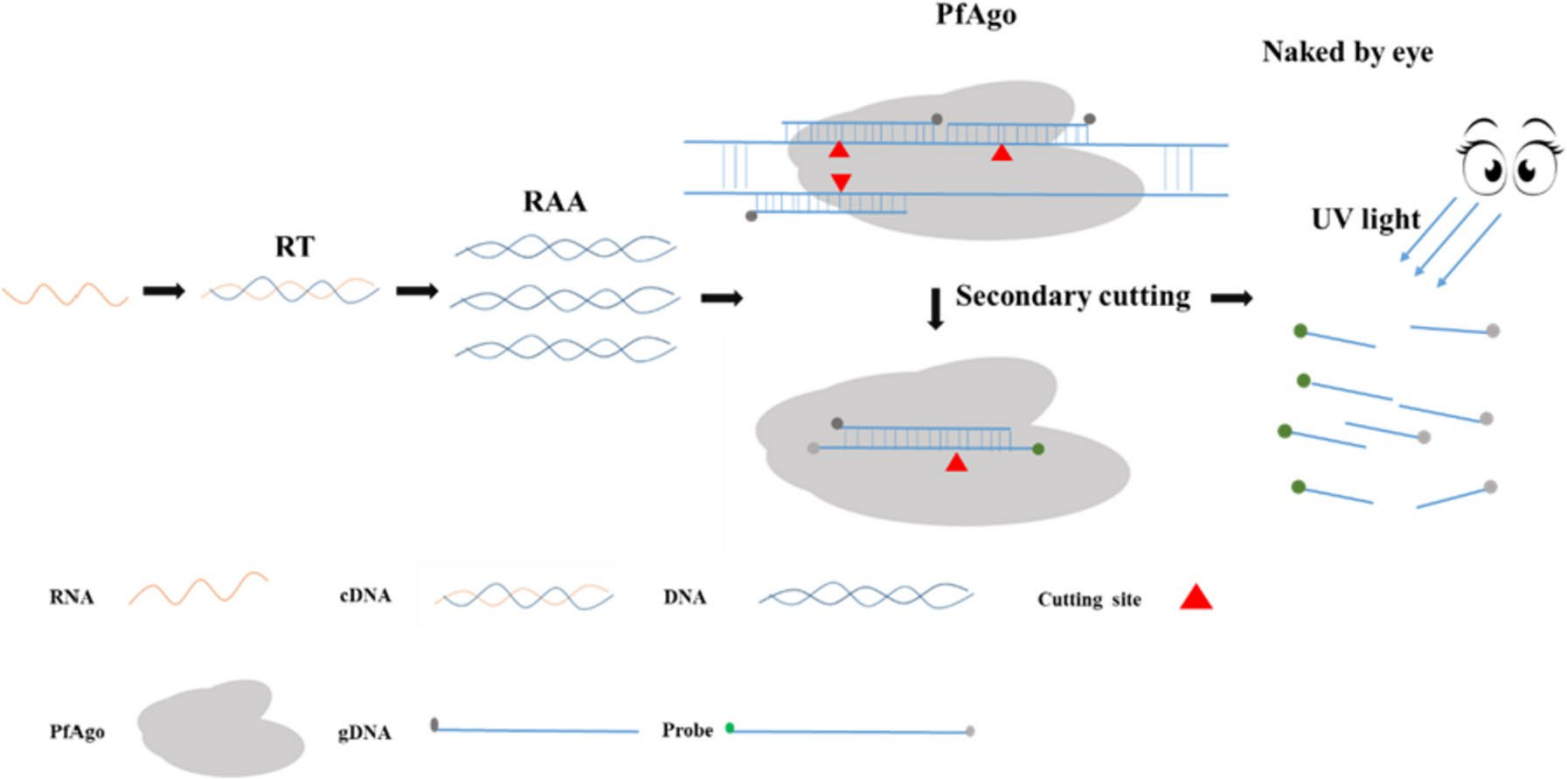
Schematic illustration of the workflow for RAA-PfAgo reaction

Recombinase-aided amplification (Wang et al. 2023) is an isothermal amplification technique that combines recombinant enzymes with other enzymes to achieve DNA or RNA amplification in vitro. In this study, the authors employed a combination of recombinase-aided amplification (RAA) and PfAgo to develop a novel detection technique for PEDV. The principle of the RAA-PfAgo process is presented in Fig. 1. In short, the PEDV RNA is isolated and then reverse-transcribed into cDNA. Subsequently, target fragments were amplified on cDNA using RAA at 39 °C for 30 min. PfAgo can specifically cleave the RAA amplified fragment strand that is complementary to the sequence of gDNA (the first cleavage) and release 16 bp of new gDNA by using the gDNAs guide, which is a set of gDNAs targeting the conserved region of the PEDV N gene (gDNA1 and gDNA2 are closely adjacent and complement to the N gene’s sense chain, while gDNA3 is complementary to the antisense chain). The newly released genomic DNA fragment possessing a 5′-phosphorylation can interact with unoccupied PfAgo proteins, resulting in the cleavage of molecular probe. The molecular probe that has been cleaved can emit fluorescence when exposed to blue light. This method can detect 100 copies of PEDV nucleic acid and has no cross-reactivity with other pathogens. The process can be completed using only two specific temperatures and does not necessitate costly equipment. This study offers significant evidence to support the effective management and containment of the PEDV outbreak.

## Methods

### The nucleic acid of viruses

TIANamp Virus DNA/RNA Kit (Tiangen, Beijing, China) was used to extract RNA or DNA from porcine epidemic diarrhea virus (PEDV), porcine deltacoronavirus (PDCoV), porcine transmissible gastroenteritis virus (TGEV), pseudorabies virus (PRV), porcine circovirus 2 (PCV2), and porcine circovirus 3 (PCV3). Then, cDNAs were synthesized with the PrimeScript RT Master Mix (Takara, Beijing, China) and stored at − 80 °C until use.

### The utilization of oligonucleotide primers for amplification, gDNAs, and probe synthesis

A set of PCR and RAA primers was developed targeting the conserved region of the PEDV N gene. PCR-F/R was used to amplify the entire N gene of PEDV, while RAA-F/R was used for RAA amplification. Additionally, a conservative group of gDNAs was also designed to cleave the double-stranded DNA of RAA products, resulting in a 16 bp single-stranded DNA for probe reporter cleavage. The primers, 5′-phosphorylated guide DNAs (gDNAs), fluorescent single-stranded DNA (ssDNA) reporter (5′6-FAM-ssDNA-BHQ1-3′), and lateral flow dipstick test reporter (5′6-FAM-ssDNA-Biotin-3′) were manufactured by Sangon Biotech, located in Shanghai, China. The primers, gDNAs, and probe reporter sequences are provided in Table 1.

**Table 1:** The sequences of primers, gDNAs, and probe utilized in this work

Name	Sequence (5′–3′)
PCR-F	ATGGCTTCTGTCAGTTTTCAGGATC
PCR-R	TTAATTTCCTGTGTCGAAGATCTCG
RAA-F	GGGTTACTAATGACAAACCCCTTTCTAAGG
RAA-R	GCAACCCAGAAAACACCCTCAGTACGAGTC
gDNA1	CGCATGCGCCAGCGAA
gDNA2	AATTCGCTCACCACGG
gDNA3	CAAATTCGCTGGCGCA
Probe	TCCACGGCGCATGCGCCTTT

### Construction of standard plasmid

In a reaction mixture of 20 μL, the components included 1 μL of template, 0.5 μL PCR-F and PCR-R (10 μM), 10 μL of 2 × Taq Plus Master Mix (Vazyme biotech co. Ltd, 137 Nanjing, China), and 8 μL of ddH_2_O. The PCR reaction was conducted with the following parameters: an initial denaturation step at 95 °C for 5 min, followed by 35 cycles of denaturation at 95 °C for 15 s, annealing at 60 °C for 15 s, and extension at 72 °C for 45 s. The entire N gene of PEDV was isolated and inserted into the pMD19-T plasmid. The concentration of the DNA was determined using a Nanodrop 2000 spectrophotometer (Thermo Fisher Scientific, Waltham, MA, USA) after being stored at − 20 °C.

### Expression and purification of PfAgo

The pET28a-His-PfAgo was chemically synthesized by Sangon Biotech (Shanghai, China) based on the nucleic acid sequence of PfAgo (GenBank accession number: OR402834). Subsequently, the manufactured construct was introduced into *E. coli* Rosetta (DE3) pLysS Singles Competent Cells (Millipore, Burlington, MA, USA) by a transformation process. Overnight cultures (10 mL) were added to 400 mL LB broth (with kanamycin, 50 μg/mL) and grown at 37 °C to an OD600 of 0.6. Isopropyl-β-D-1-thiogalactopyranoside (IPTG, 1 mM) was incorporated into the culture medium, and induction was carried out at 16 °C and 180 rpm for 20 h. The supernatant protein was purified using a His-tag Protein Purification Kit (Sangon Biotech, Shanghai, China) after the cells were centrifuged at 15,000 g for 20 min at 4 °C. This study employed a 50 KD ultrafiltration column (Millipore, Darmstadt, Germany) to concentrate the pure target protein. Subsequently, the identification of the purified protein was conducted using SDS-PAGE.

### The RAA reaction

RAA reaction mixture (ZC Bio-Sci &Tech Co. Ltd, Hangzhou, Zhejiang, China) containing primer RAA-F (10 μM, 2 μL), primer RAA-R (10 μM, 2 μL), buffer A (25 μL), ultrapure water (13.5 μL), buffer B (2.5 μL), and template DNA or cDNA (5 μL) was used to carry out the RAA reaction. Furthermore, to achieve the most favorable reaction conditions for the RAA process, a series of experiments were conducted at various temperatures (37, 38, 39, 40, 41, and 42 °C) and reaction durations (0, 20, 30, 40, and 50 min). The temperature and duration of the RAA reaction were controlled using PTC-200 thermal cyclers manufactured by MJ Research in Waltham, MA, USA. The resulting compounds were subsequently identified through the utilization of agarose gel electrophoresis.

### The PfAgo reaction

The PfAgo reaction system (20 μL) which includes 2 μM PfAgo, 0.25 μM each gDNA (total three gDNAs), 0.5 μM probe, 0.5 mM Mncl_2_, and 10 μL RAA production. The tube was placed in PTC-200 Thermal cyclers (MJ Research, Waltham, MA, USA) at 95 °C for 30 min, and the fluorescence signal following the PfAgo-mediated cleavage reaction was observed under blue light.

### RAA-PfAgo detection system evaluation

Determination of analytic sensitivities of RAA-PfAgo detection systems was carried out by using pMD19-PEDV-N DNA ranging from 1 × 10^4^ to 1× 10^1^ copies/μL and the RAA-PfAgo sensitivity reaction was conducted through PTC-200 Thermal cyclers (MJ Research, Waltham, MA, USA) or real-time fluorescence quantitative PCR instrument (CFX96TM, Bio-Rad, Hercules, CA, USA) at 95 °C for 30 min. The genomic cDNA or DNA of PRV, PCV2, PCV3, PEDV, TGEV, and PDCoV were utilized to test the detection system’s specificity, and the result was detected under blue light after 30 min of the RAA-PfAgo reaction at 95 °C.

### The RAA-PfAgo-LFD reaction

The RAA-PfAgo-LFD reaction system (20 μL) including 2 μM PfAgo, 0.25 μM each gDNA (total three gDNAs), 125 nM probe, 0.5 mM Mncl_2_, and 10 μL RAA production. For 30 min, the tube was placed in PTC-200 Thermal cyclers (MJ Research, Waltham, MA, USA) at a temperature of 95 °C. After the RAA-PfAgo reaction, 30 μL of ultrapure water was added to the reaction tube, mixed well, and a test strip (JY030, Tiosbio, Beijing, China.) was inserted into the tube, followed by observing results after 5 min.

### Identification of PEDV in clinical samples

Newborn piglets with diarrhea were collected from seven farms in Sichuan, China, yielding 53 clinical specimens (including feces and intestinal samples). Total RNAs were extracted from these samples and then reverse-transcribed to cDNA, serving as templates in PCR (Tian et al. 2021) or RAA-PfAgo/RAA-PfAgo-LFD assay.

## Results

### PfAgo expression and purification outcomes

**Fig. 2:**
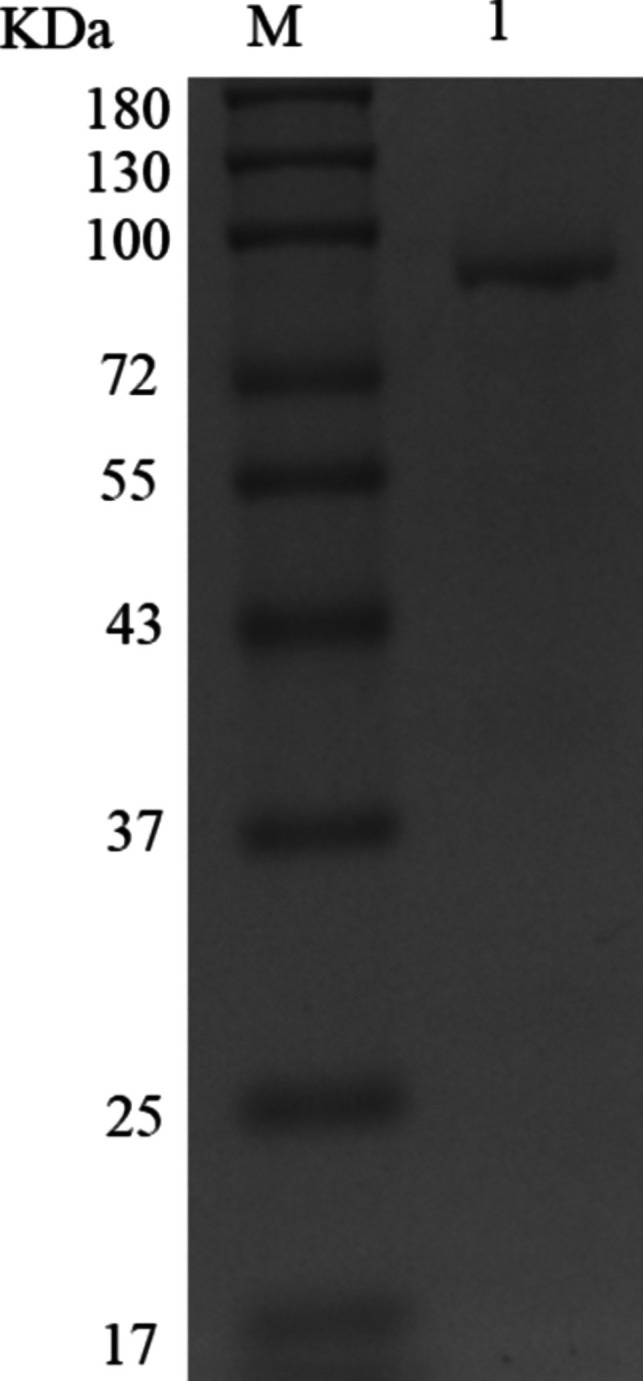
Sodium dodecyl sulfate-polyacrylamide gel electrophoresis (SDS-PAGE) analysis of purified PfAgo. M, marker; 1, PfAgo

The PfAgo protein expression was induced by 1 mM IPTG for 20 h at 16 °C. The pellets were resuspended after harvesting, then lysed, and finally concentrated. The expressed PfAgo (about 87 KDa) was dissolved primarily in the supernatant and eluted by elution buffer (Fig. 2).

### The results of optimization of the RAA reaction

**Fig. 3:**
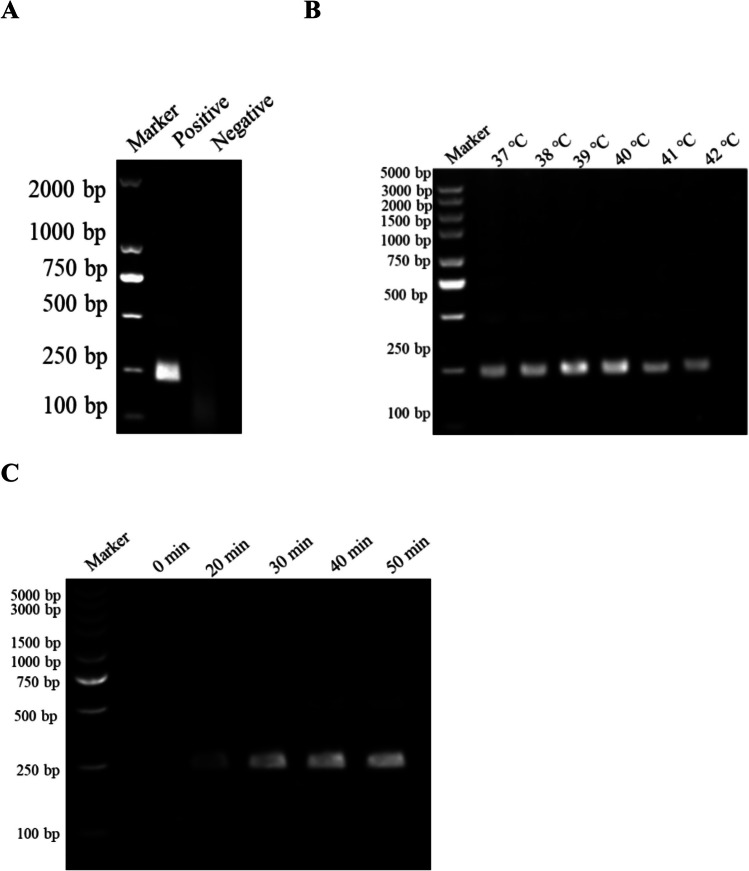
Optimization of the RAA assay. **A** The RAA assay. **B** Assessment of different incubation temperatures (37 °C, 38 °C, 39 °C, 40 °C, 41 °C, and 42 °C). **C** Assessment of different incubation times (0, 20, 30, 40, and 50 min)

ddH_2_O and 1 × 10^5^ copies/μL of the pMD19-PEDV-N DNA were used as the RAA template. After 30 min of RAA reaction at 39 °C, there were specifically sized bands in PEDV-positive DNA but none in the negative control (Fig. 3A), demonstrating the efficacy of the RAA primers (RAA-F/R). To enhance the efficiency of the RAA reaction, the reaction was conducted at various temperatures and durations to explore optimal conditions. According to the data, the optimum conditions for the RAA reaction were achieved at a temperature of 39 °C (Fig. 3B) and a reaction period of 30 min (Fig. 3C).

### The results of the RAA-PfAgo detection system

**Fig. 4:**
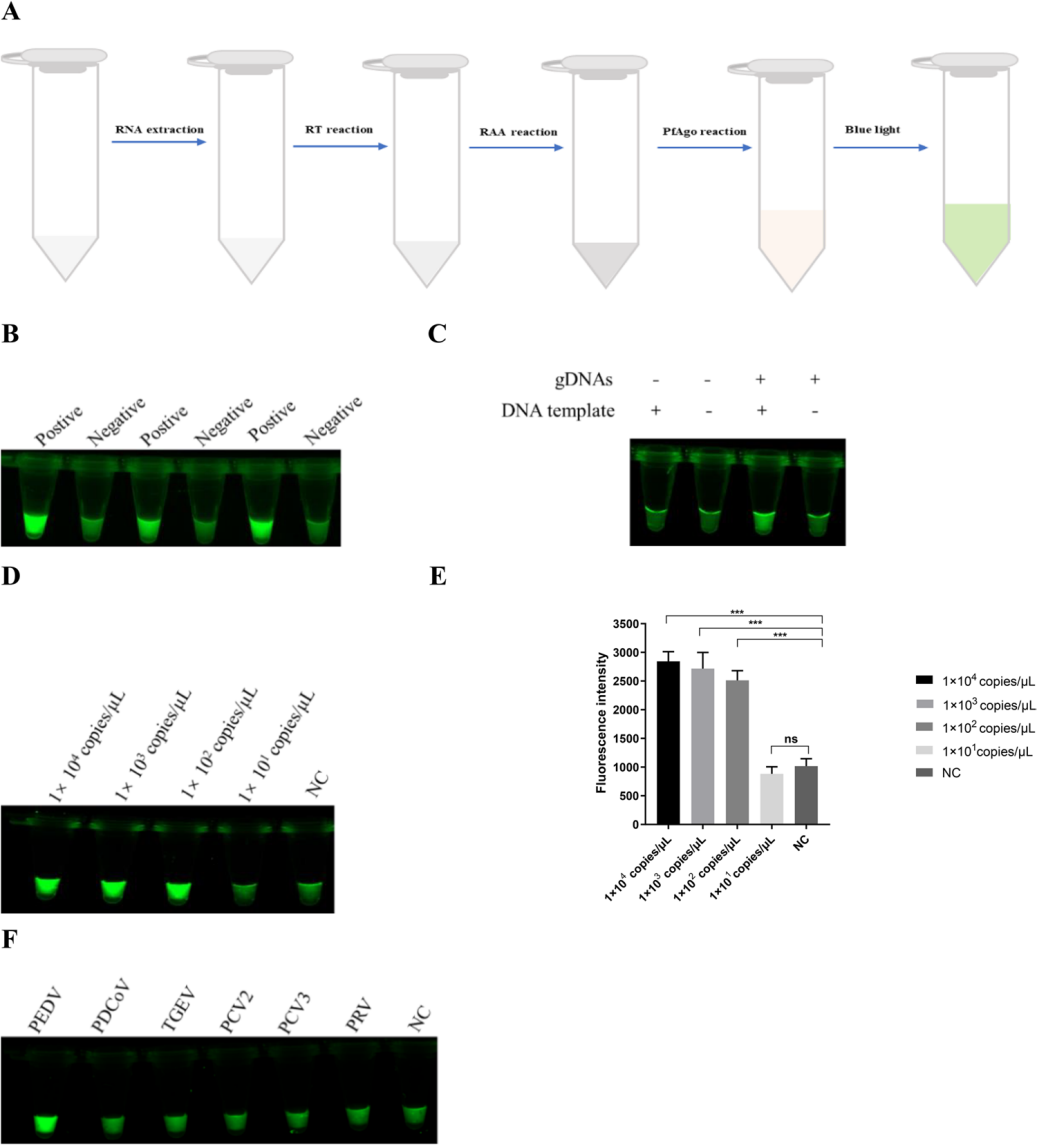
Establishing the RAA-PfAgo assay. **A** Schematic representation of RAA-PfAgo assay to detect PEDV. **B** The outcome of using blue light to identify PEDV via the RAA-PfAgo reaction. **C** RAA-PfAgo reaction outcomes when gDNAs and templates are present at the same time. **D** The sensitivity of the RAA-PfAgo to detect PEDV under blue light. The detection limit of the RAA-PfAgo assay was determined by testing 10-fold subsequent dilutions of the DNA standard over a range of 10^4^ to 10 copies/μL. **E** The sensitivity of the RAA-PfAgo to detect PEDV by real-time fluorescence detection, the detection limit of the RAA-PfAgo assay was determined by testing 10-fold successive dilutions of the DNA standard over a range of 10^4^ to 10 copies/μL (*n* = 3 replicates; error bars represent SD. ***p < 0.001, two-sample *t*-test). **F** The RAA-PfAgo assay’s specificity for identifying PEDV in blue light. NC, negative control

PfAgo and RAA were combined to increase the sensitivity of PfAgo detection. The procedure is as follows: reverse transcription (RT) of the extracted viral RNA, amplification of the obtained cDNA by RAA at 39 °C for 30 min, mixing of RAA product with PfAgo, reacted at 95 °C for 30 min, and results were observed under the blue light (Fig 4A). For the RAA-PfAgo detection system, 1 × 10^5^ copies/μL of the pMD19-PEDV-N DNA and ddH_2_O was used as the RAA template, and the amplification products were added to the PfAgo system to measure fluorescence value. This study’s findings demonstrate a significant increase in positive fluorescence values compared to negative fluorescence values when exposed to blue light (Fig. 4B). Additionally, the results depicted in Fig. 4C indicate that PfAgo could cleave the probe and generate fluorescence signals only in the presence of both gDNAs and templates. Based on these observations, it can be concluded that the RAA-PfAgo system can be utilized to detect PEDV. The sensitivity of the RAA-PfAgo reaction was evaluated using a 10-fold serially diluted template ranging from 1 × 10^4^ to 1× 10^1^ copies/μL of the pMD19-PEDV-N DNA. The obtained findings demonstrated the potential of the developed detection system to detect as low as 1 × 10^2^ copies/μL of the dsDNA template (Fig. 4D) under blue light. This observed limit of detection (LOD) was consistent with the value detected by real-time fluorescence detection (Fig. 4E). Figure 4F demonstrates the capability of the RAA-PfAgo detection system to detect PEDV and no cross-reaction with other viruses. The results suggest that the established RAA-PfAgo detection method for PEDV has high sensitivity, specificity, and reproducibility.

### The results of the RAA-PfAgo-LFD reaction

**Fig. 5:**
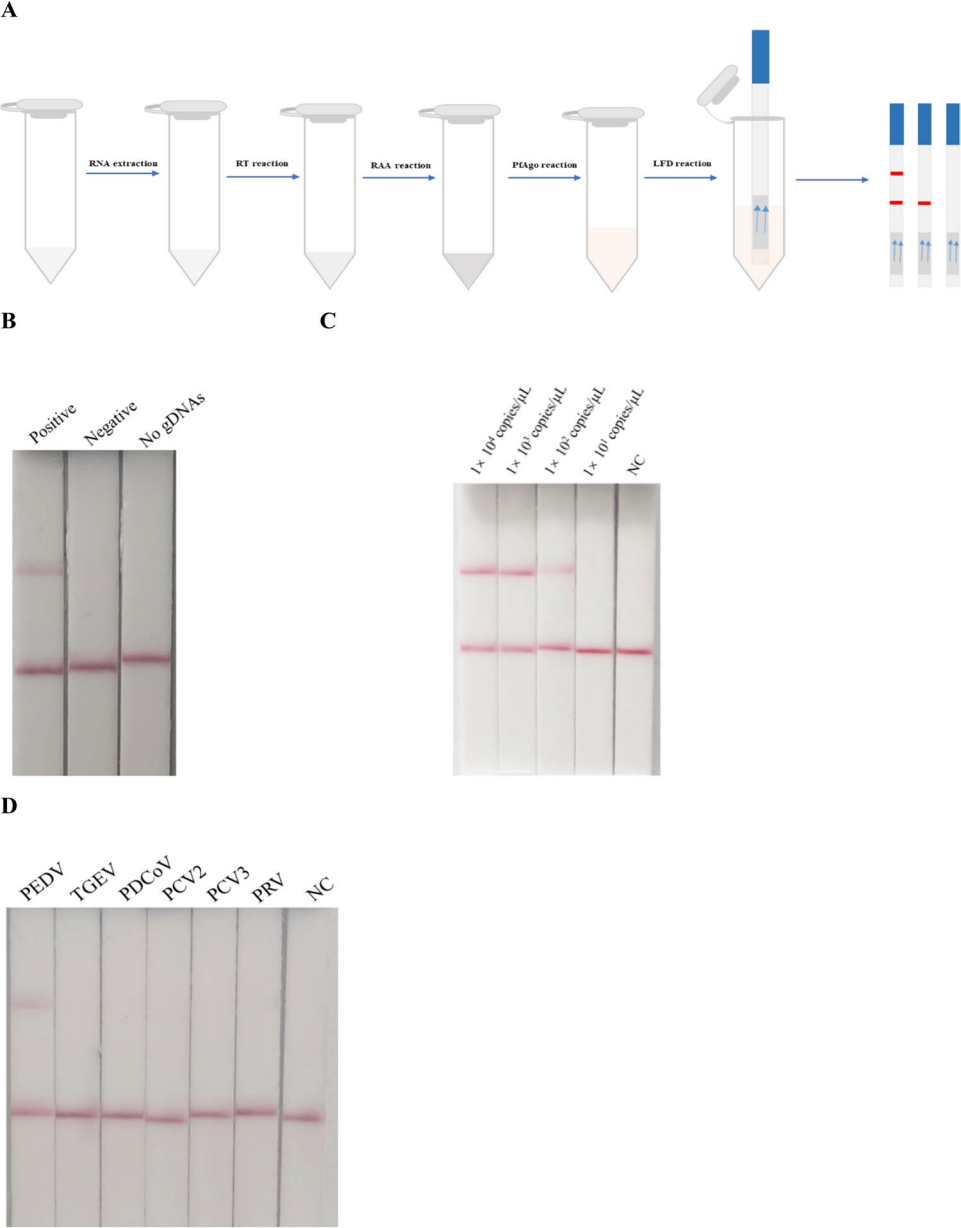
Establishing the RAA-PfAgo-LFD. **A** Schematic illustration of RAA-PfAgo-LFD assay to detect PEDV. **B** The result of the RAA-PfAgo-LFD reaction to detect PEDV. **C** The assay’s sensitivity in detecting PEDV using the RAA-PfAgo-LFD method. **D** The assay’s specificity in detecting PEDV using the RAA-PfAgo-LFD method. NC, negative control

In the RAA-PfAgo-LFD method, the RNA of the virus was isolated and subsequently converted into cDNA using reverse transcription. The resulting cDNA was then used for amplification using the RAA technique, which was carried out at 39 °C for 30 min. The RAA product was added to the PfAgo system and allowed to react at 95 °C for 30 min, followed by an LFD reaction. If the 5′-6-FAM-ssDNA-Biotin-3′ reporter gene was utilized, the FAM modification group binds to the gold NP anti-FAM antibody, forming a complex caught by the antibody detection line. A chain avidin (control band) line can capture biotin-modified groups. Bands are seen exclusively at the control line when PfAgo did not destroy ssDNA reporter molecules and are captured by the streptavidin system. After the PfAgo-mediated degradation of the ssDNA reporter, although the streptavidin line (control band) captured the biotin-modified group owing to the cutoff of the ssDNA reporter molecule, the broken FAM-modified group can also be captured by the antibody detection line, resulting in the appearance of bands on both the control and the detection lines (Fig. 5A). As demonstrated in Fig. 5B, it can be observed that both the detection and control lines of the positive sample exhibited bands. However, only the control line of the negative ddH_2_O or no gDNAs control presented a band. This observation suggests that the developed RAA-PfAgo-LFD technique is feasible. The LOD of the RAA-PfAgo-LFD for PEDV was 1 × 10^2^ copies/μL (Fig. 5C), and specific tests suggested that the method cannot detect other pathogens (Fig. 5D).

### The results of RAA-PfAgo/RAA-PfAgo-LFD to detect PEDV in clinical samples

A total of 53 typical clinical samples were successfully evaluated (nine were identified as PEDV positive by the PCR) to establish the validity of RAA-PfAgo tests. The RAA-PfAgo and RAA-PfAgo-LFD PEDV detection rates were consistent with the PCR (Table 2).

**Table 2:** A comparative analysis of RAA-PfAgo/RAA-PfAgo-LFD and standard PCR techniques in clinical samples

Samples type	Number of samples	RAA-PfAgo		RAA-PfAgo-LFD		Conventional PCR	
		Positive	Negative	Positive	Negative	Positive	Negative
Fecal swab	11	2	9	2	9	2	9
Intestinal	42	7	35	7	35	7	35
Total	53	9	44	9	44	9	44

## Discussion

PEDV is a highly contagious swine enteric infection that results in significant economic losses for the pig industry (Li et al. 2021a). Despite inactivated or attenuated vaccinations being used to combat PEDV (Li et al. 2017), PEDV is still prevalent worldwide, resulting in enormous economic losses to farmers. China has a large pig farming industry. Although agriculture is increasingly mechanized, not all farms have professional virus detection equipment and personnel. Virus detection requires the shipment of collected samples to professional laboratories, which frequently wastes significant human and material resources. Currently, the majority of available methods for PEDV surveillance have numerous drawbacks, such as insensitivity and unsuitability for field detection. Therefore, a simple, convenient, and fast detection method is urgently required for PEDV detection.

Isothermal amplification technology is a recent detection method that includes loop-mediated isothermal amplification (LAMP) (Yu et al. 2015), cross primer amplification (CPA) (Qiao et al. 2015), rolling circle amplification (RCA) (Zhang et al. 2021), and recombinase polymerase amplification (RPA). The limitations of LAMP (Kim et al. 2021; Li et al. 2022) include time-consuming primer design and vulnerability to nonspecific amplification. CPA (Frączyk et al. 2016) requires multiple primers, increasing reaction cost and complexity. One limitation of the RCA (Zhu et al. 2023) technique is its requirement for circular DNA as a template. In contrast, it should be noted that RPA (Wang et al. 2017) exhibits a notable degree and effectiveness in amplification. Furthermore, it is worth mentioning that RPA does not necessitate intricate primer design and can extract a greater quantity of target nucleic acids within a comparatively brief duration. However, because RPA is susceptible to false positives, it is frequently necessary to combine RPA and CRISPR systems to increase the specificity of the detection (Kellner et al. 2019; Wu et al. 2022). Currently, there are numerous detection methods for the combination of RPA and CRISPR (Chen et al. 2022a; Lei et al. 2022), but CRISPR has its limitations, such as the necessity for crRNA guidance to cut target nucleic acids or its sensitivity to high temperatures, which limits its application in testing.

When comparing CRISPR to PfAgo, it is evident that PfAgo exhibits the advantage of requiring a smaller amount of gDNA to cleave the target sequence. Furthermore, PfAgo and gDNA resist elevated temperatures, making PfAgo a more appropriate choice for regions with limited and distant resources (He et al. 2019; Swarts et al. 2015; Wang et al. 2021). In recent years, PfAgo-based detection methods such as PCR combined with PfAgo (He et al. 2019), LAMP combined with PfAgo (Xun et al. 2021), and RPA combined with PfAgo have emerged progressively (Yang et al. 2023; Zhao et al. 2022). Previous studies have described using RPA and PfAgo to detect enterocytozoon hepatopenaei (Yang et al. 2023). Although the technique exhibits exceptional specificity and sensitivity, including the PfAgo reaction after the purification of the RPA amplification product inevitably introduces a higher level of intricacy to the overall procedure. In our research, we focused on RAA because PCR requires complex instruments and a long time, while LAMP is quite challenging in constructing primers. RAA, similar to RPA, has the advantages of rapid nucleic acid amplification at room temperature and simple operation (Wu et al. 2022). However, due to the need for purification, nucleic acid electrophoresis, and the tendency for false positives to occur when using RAA alone (Chen et al. 2022b; Wang et al. 2023), we combine PfAgo with RAA to increase the convenience and specificity of detection based on the high sensitivity and specificity of PfAgo cleavage activity. In using RAA and PfAgo, a visual detection approach was developed for PEDV in this article. To minimize the possibility of false negative, we developed primers and gDNA based on the conservative PEDV N gene. Furthermore, the enhancement of RAA amplification efficiency and specificity was achieved by optimizing amplification settings. To streamline operations and minimize environmental pollution, the RAA product obtained from the reaction at 39 °C for 30 min was directly transferred to the PfAgo system without any purification steps. Subsequently, the transferred product was heated at 95 °C for an additional 30 min. The analysis of the reaction outcomes involved subjecting the substance to blue light. The detection strategy significantly benefits from its simplicity and versatility since it requires heating and blue light equipment. In order to enhance the examination of the outcomes, the RAA-PfAgo reaction was integrated with LFD (lateral flow dipstick) and assessed for its specificity, sensitivity, and practicality in the implemented approach. According to the results, RAA-PfAgo and RAA-PfAgo-LFD could detect 100 copies of viral nucleic acid without cross-reactivity with other organisms. Clinical samples were then utilized to assess the method’s dependability, which was compatible with the PCR results.

In conclusion, a simple, portable, economical, and highly sensitive detection platform was developed for PEDV. This technique provides new technical support for detecting and preventing PEDV without requiring precise instruments or complex experimental procedures.

## Data Availability

The data presented in this study are available on request from the corresponding author.
